# Development and analysis of acceptance of a nutrition education package among a rural elderly population: an action research study

**DOI:** 10.1186/1471-2318-12-24

**Published:** 2012-06-07

**Authors:** Suzana Shahar, Siti Nur’Asyura Adznam, Suriah Abdul Rahman, Noor Aini Mohd Yusoff, Zaitun Yassin, Fatimah Arshad, Noor Ibrahim Mohamed Sakian, Mohmad Salleh, Asnarulkhadi Abu Samah

**Affiliations:** 1 Dietetics Programme, Faculty of Health Sciences, Universiti Kebangsaan Malaysia, Jalan Raja Muda A., Aziz 50300, Kuala Lumpur, Malaysia; 2 School of Chemical Sciences and Food Technology, Faculty of Sciences and Technology, Universiti Kebangsaan Malaysia, Bandar Baru BangiBangi, Selangor, Malaysia; 3 Faculty of Therapeutic Sciences, Masterskill University College of Health Sciences, Cheras, Selangor, Malaysia; 4 Department of Nutrition and Dietetics, Faculty of Medicine and Health Sciences, Universiti Putra Malaysia, Serdang, Selangor, Malaysia; 5 Department of Dietetics, International Medical University, Bukit Jalil, Selangor, Malaysia; 6 Occupational Therapy Programme, Faculty of Health Sciences, Universiti Kebangsaan Malaysia, Kuala Lumpur, Malaysia; 7 Family Health Development Division, Ministry of Health Malaysia, Putrajaya, Malaysia; 8 Department of Social and Development Science, Faculty of Human Ecology, Universiti Putra Malaysia, Serdang, Selangor, Malaysia

**Keywords:** Elderly, Rural population, Education models, Health promotion, Nutrition therapy

## Abstract

**Background:**

It is well known that older adults are often vulnerable to malnutrition. This action research was conducted to develop a nutrition education package for promoting healthy ageing and reducing risk of chronic diseases among older adults in a rural area of Malaysia.

**Methods:**

This study was designed and conducted in three stages, including needs assessment, development of the package and analysis of acceptance among 33 older adults aged 60 years and over in rural communities, and 14 health staff members at rural health clinics. Subjects completed a questionnaire including sociodemographic factors and acceptance evaluation of the nutrition education package with respect to content, graphics and design. Data were analysed descriptively using numbers and percentages.

**Results:**

A nutrition education package comprising a booklet, flipchart and placemats was developed. A total of 42.4% of the older adults expressed that the sentences in the flipchart needed to be simplified and medical terms explained. Terminology (60%), illustrations (20%) and nutrition recommendations (20%) were the aspects that prevented elderly subjects from fully understanding the booklet. Information on the placemats was easily understood by subjects.

**Conclusions:**

A well accepted nutrition education package for promoting healthy ageing and reducing risk of chronic diseases was developed that incorporated modifications based on feedback from older adult subjects and health clinic staff in a rural area. It is a tool that can effectively be used for health education in this population.

## Background

Tackling nutritional issues among older adults in Malaysia is a challenge because of both undernutrition and overnutrition, with half of these adults being illiterate [1]. Within a decade, the prevalence of overweight among Malaysian older people doubled from 15.6% in 1996 to 29.8% in 2006. Obesity increased more than three-fold from 3.1% in 1996 to 10.9% in 2006. However, the prevalence of overweight decreased with age from 35.63% (age 60-64 years) to 12.64% (80+ years). A similar trend has been noted for obesity [1]. As in other rapidly developing countries, the prevalence of chronic diseases among the Malaysian population is on the rise, with the highest prevalence among those age 50 years and older [2,3].

Lifestyle and dietary changes should be advocated to curb the rise of such diseases. However, the nutritional knowledge of older adults [4] in rural areas is inadequate [5,6]. A recent study among adults with diabetes at a government health clinic [7] indicated that older adults had lower nutritional knowledge scores than others. Illiteracy is widespread but often a hidden problem, and those with the lowest literacy rates also have the poorest health status. These facts must be considered when developing and evaluating suitable educational print materials for patients and their families [8]. Therefore, development of an appropriate nutrition education package could be effective in improving the quality of dietary intake and lifestyle of older adults in Malaysia. The Family Health Division of the Ministry of Health of Malaysia [9] has developed a series of educational materials, including some for older adults. However, a nutrition component is plainly lacking.

Thus, the aim of this study was to develop a nutrition education package about healthy ageing and reducing the risk of chronic diseases for implementation at health clinics in a rural area of Malaysia. This is an action research study with the aim of facilitating change in a particular community and program. Previously, authors reported the needs assessment findings from this study [10]. This manuscript will present the developmental aspects of the package and analysis of its acceptance among health professionals and older people. We hypothesised that an education package that was developed based on needs of the community would be well accepted by both health professionals and older adults. This study was approved by the Universiti Kebangsaan Malaysia Medical Research and Ethics Committee.

## Methods

The study was designed and conducted in three stages, as shown in Table 1. In each stage, specific data collection techniques were employed to elicit facts and gather data from various sources of information such as documents, guidelines, protocols and targeted respondents. As in other action research, involvement of the respondents in developing the product was essential. In this study, older adults were involved as partners [11] in developing and evaluating the nutrition education package, representing the underpinning philosophy held by the research team members. 

**Table 1:** Stages, data collection technique and sources of information

**Stage**	**Activity**	**Data collection method & technique**	**Source of information**
I	Developing nutritional education package	· Content analysis	Manual, guideline, protocol
II	Evaluating of acceptance towards nutrition education package	· Survey using questionnaire	Elderly people & health staff
		· Personal evaluation (critique)	
III	Finalise on the ‘adjusted’ nutrition education package	· Personal evaluation (critique)	Elderly people & health staff

### Development of flipchart, booklet and placemats

An extensive literature review on available nutrition education packages and dietary guidelines in Malaysia including the Malaysian Dietary Guidelines [12], Module on Skills for Healthy Eating [9], Guide for Elderly People in Institutions [9], Guide for Nutrition in Elderly People [9] and Guide for Caregivers of the Elderly [9] as well as results from the previous needs assessment study among rural elderly Malays [10] was conducted to develop a suitable nutrition education package. Nutrition guidelines for older people from other countries including Dietary Guidelines for Older Australians [13], Dietary Guidelines for Americans, Education Resource Packet [14], Dietary Guidelines for Healthy American Adults [14], Nutrition Guidelines for Britain [15] and the Nutrition Guide for South Africa [16] were also evaluated. At this stage, a content analysis approach was employed to study and examine the various related documents. The information gathered became the basis for developing the content of the package concurrent with consideration of nutritional and health problems as well as dietary habits and lifestyles of Malaysian older adults.

A professional artist was employed to design illustrations that would assist in local older adults’ understanding of nutrition information. Photos of foods and meals were captured in a photography studio. A series of meetings with the research group comprising dietitians, nutritionists, public health physicians and an anthropologist were conducted to finalise the content, graphics and design of the educational package as well as its suitability with regard to the multi-ethnic background of Malaysian society.

### Evaluation of the nutrition education package

The nutrition education package was evaluated by older adults aged 60 years and over who were able to read and write, had no hearing or sight disabilities and no mental or terminal illnesses. It was also evaluated by health staff who had been involved in the care of elderly people for the past year. A total of 33 elderly subjects (12 men and 20 women), all of whom had a formal education, participated in this study. The staff consisted of physicians (n = 3), medical assistants (n = 3) and nurses (n = 8), with a mean age of 30.9 ± 8.3 years. Their education level included a higher school certificate (n = 4), diploma (n = 7), undergraduate degree (n = 2) or masters degree (n = 1). Both older adults and health staff were recruited from two health clinics in Klang Valley of Malaysia. Evaluation sessions were carried out separately for older adults and health staff.

Subjects were asked to complete a self-administered questionnaire consisting of sociodemographic parameters and acceptance evaluation of the nutrition education package with respect to content, graphics and design as suggested by Hawe et al. [17]. Subjects were also asked to indicate their preference for the newly developed ‘food plate’ compared with the present food pyramid [12]. They were given ample time (i.e., approximately 45 to 60 minutes) in a designated room to review the education package.

Fieldworkers were available nearby if subjects needed any clarification or assistance.

## Results

### Education package

**Figure 1:**
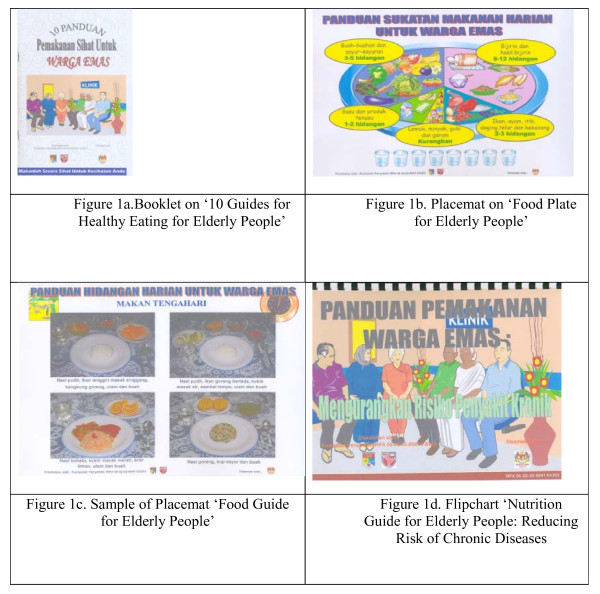
Nutrition education package for healthy aging.

Based on the needs assessment exercise in phase 1 reported earlier [10] and the extensive literature review, a nutrition education package consisting of three modes of learning (i.e., a booklet, flipchart and placemats) (Figure 1) was developed.

### Booklet

A booklet entitled ‘10 Healthy Eating Guidelines for Older Individuals’ was developed (Figure 1a) that included information on sensible dietary practices for achieving healthy ageing, suitable for older adults who were able to read or for their caregivers and health professionals. It consisted of a ten-point guide to healthy eating and lifestyle including notes on food safety, as shown in Table 2.

**Table 2:** Title content of booklet and flipchart

**Booklet (10 messages)**	**Flipchart (6 guides)**
Message 1: Take a variety of food	What is obesity?
	Guide 1: Weight management
Message 2: Be physically active for muscle Strength	What is hyperlipidemia?
	Guide 2: To reduce fat & cholesterol
Message 3: Take at least 3 main meals in a day	What is hypertension?
	Guide 3: To control Blood Pressure
Message 4: Increase the consumption of fruits and vegetables	What is Diabetes Mellitus?
	Guide 4: To control Blood Sugar
Message 5: Meet your calcium requirement	What is Fiber?
	Guide 5: To increase fiber in diet
Message 6: Reduced intake of foods high in fat and cholesterol	Guide 6: Exercise for older people
Message 7: Reduced salt in cooking and foods high in sodium content	
Message 8: Reduced sugar and foods high in sugar	
Message 9: Drink plenty of water	
Message 10: Safe food handling	

The booklet began with an illustration of physiological changes with ageing and their implications for health and nutritional requirements. Message 1 in the booklet conveyed the importance of eating a variety of foods, using a new concept (i.e., food plate) instead of the conventional Malaysian food pyramid [12]. The food plate is a modification of the food pyramid, with the addition of a water intake recommendation of up to 8 glasses per day and a change in serving size of proteins suitable for elderly individuals [18]. This new concept was evaluated for acceptance in this study.

### Placemats

In view of the low literacy level of the studied population, message 1 ‘eat a variety of foods’ was extracted from the booklet and used to develop a placemat ‘Food Plate: Daily Food Portions for Older People’. The food plate indicated serving sizes of foods from the main food groups, including carbohydrates, proteins, fats, vegetables and fruits (Figure 1b). The other four placemats had a photographic sample menu of breakfast, lunch, snacks or dinner and were titled 'Daily Food Guide for Older People'. Four samples of menus were provided for each meal time. To provide a guide for meal times, an illustration of a clock and common events surrounding each meal time of the day (i.e., breakfast, lunch, dinner and snacks) were inserted at the corner of each placemat (Figure 1c). Placemats were A3 paper sized (420 mm × 297 mm) and illustrated with appropriate graphics and food photos, suitable to be used on the dining table to remind older adults about healthy food choices and portion sizes.

### Flipchart

To provide information on reducing risk of chronic diseases, a flipchart entitled ‘Nutrition Guide for Older People: Reducing Risk of Chronic Diseases’ was developed. This flipchart could be used by health professionals to provide dietary advice for older adults about conditions related to metabolic disturbances including obesity, diabetes mellitus, hypertension, hyperlipidaemia and cardiovascular disease (Figure 1d). It could be used during individual or group counselling. It listed six guidelines for reducing risk of chronic diseases, with 58 A5 size (210 mm x 148 mm) pages (Table 2). Each nutrition education point was preceded with an introduction about the health problem or disease followed by a nutrition recommendation, with illustrations that facilitated comprehension in low literacy individuals. Notes for the educator were outlined on the back of each flipchart page as a detailed guide for dietary advice or counselling.

### Acceptance of nutrition education package

Assessment of acceptance involved 33 older adult subjects aged 60 to 79 years (mean age 67.1 ± 4.8 years), with 64% men. Most of the older subjects were Malays (91%), followed by Chinese (6%) and Indians (3%). In addition, 14 health staff aged 30.9 ± 8.3 years (23 to 50 years; 79% women) also volunteered to participate. They were nurses (57%), medical officers (22%) and assistant medical officers (21%).

### Flipchart

**Figure 2:**
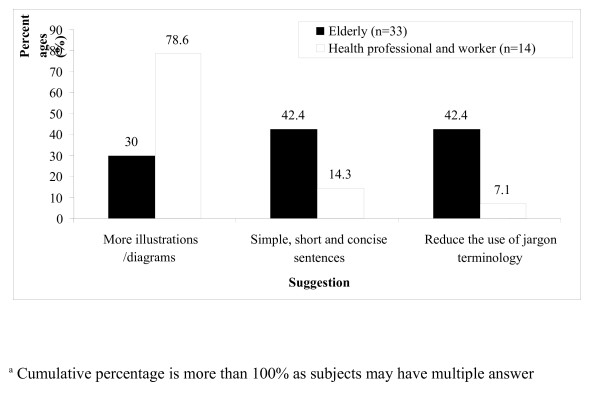
Suggestions to improve understanding towards the flipchart.

Seventy-nine percent of the older adult subjects reported that they understood the information in the flipchart (Table 3). The majority reported that appropriateness of the recommendations (67%) and clearly understood sentences (61%) were aspects that facilitated their understanding. However, only 46% reported that they understood the medical terms used in the flipchart. Approximately 42% of the older adult subjects expressed that sentences needed to be simplified, and some of the terminology (e.g., obesity, hyperlipidaemia, hypertension, diabetes mellitus and fibre) needed to be reduced or explained (Figure 2). All health professionals reported that they understood the information in the flipchart (Table 4). However, some suggested that more illustrations be included (79%).

**Table 3:** Analysis of acceptance of flipchart among elderly and health staff subjects [Number (%)]

**Parameters**	**Elderly subjects**			**Health staff (n = 14)**
	**Men (n = 21)**	**Women (n = 12)**	**Total (n = 33)**	
Understanding on information:				
Understood	17 (81.0)	9 (75.0)	26 (78.8)	(100.0)
Not or less understood	4 (19.0)	3 (25.0)	7 (21.2)	0 (0.0)
Aspects facilitate comprehension:^a^				
Terminology easily understood	10 (58.8)	5 (55.6)	15 (45.5)	13 (92.9)
Sentences clear and easily understood	13 (76.5)	7 (77.8)	20 (60.6)	10 (71.4)
Figures clear, suitable and attractive	9 (52.9)	8 (88.9)	17 (51.5)	13 (92.9)
Suitable of recommendation	14 (82.4)	8 (88.9)	22 (66.7)	9 (64.3)
Suitability of figures/illustrations				
Yes	20 (95.2)	11 (91.7)	31 (93.9)	14 (100.0)
No	1 (4.8)	1 (8.3)	2 (6.1)	0 (0.0)
Combination of colour:				
Attractive	20 (95.2)	11 (91.7)	31 (93.9)	14 (100.0)
Less/No	1 (4.8)	1 (8.3)	2 (6.1)	0 (0.0)
Font Size:				
Easy to read	21 (100.0)	11 (91.7)	32 (97.0)	14 (100.0)
Difficult to read	0 (0.0)	1 (8.3)	1 (3.0)	0 (0.0)

^c^cumulative percentages more than 100% due to multiple answers.

**Table 4:** Analysis of acceptance towards information in the booklet among elderly and health staff subjects [Number (%)]

**Assessment parameters**	**Elderly**			**Health staff (n = 14)**
	**Men (n = 21)**	**Women (n = 12)**	**Total (n = 33)**	
Undestanding of information:				
Yes	13 (61.9)	10 (83.3)	23 (69.7)	14 (100.0)
Less/No	8 (38.1)	2 (16.7)	10 (30.3)	0 (0.0)
Aspects facilitate comprehension^a^:				
Terminology easily understood	9 (42.9)	6 (50.0)	15 (45.5)	10 (71.4)
Sentences clear and easily understood	8 (38.1)	8 (66.7)	16 (48.5)	13 (92.9)
Figures clear, suitable and actractive	9 (42.9)	8 (66.7)	17 (51.5)	13 (92.9)
Tables easily undestood	7 (33.3)	7 (58.3)	14 (42.4)	11 (78.6)

^a^Cummulative percentages more than 100% due to multiple answers.

Most of the older adult subjects indicated that illustrations in the flipchart were suitable (94%) and colourful (94%) and that the font size was readable (97%). All the health staff agreed that the flipchart was acceptable with respect to illustrations, colour and font size (Table 4).

### Placemats

Both older adult subjects (94%) and health staff (100%) agreed that messages on the placemats were easily understood. Sample menus displayed on the mats were perceived as suitable by both older adults (88%) and health staff (100%). When asked about the preferred concept for understanding healthy eating, 70% of the older adult subjects chose the food plate (new concept), and 57% of the health staff preferred the food pyramid (conventional concept).

### Booklet

Most of the older subjects (70%) indicated that they understood information in the booklet (Table 4). However, a proportion of them expressed that they did not understand fully (24%) or at all (6%) the content. On the other hand, all health staff understood the information in the booklet.

**Figure 3:**
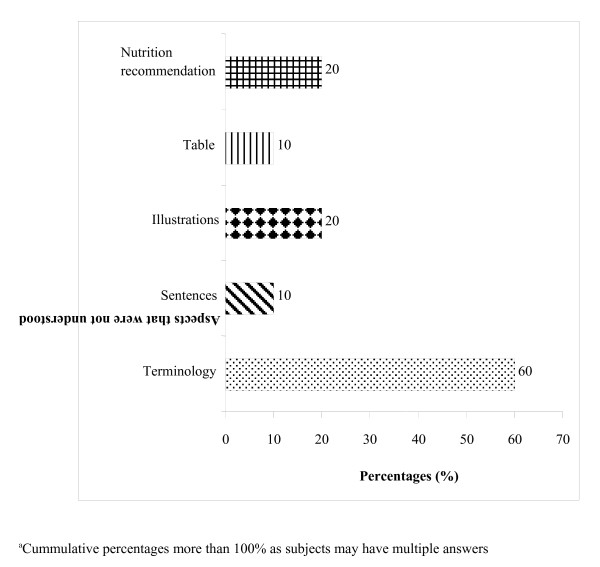
Aspects that were not understood by elderly subjects in the booklet (n = 10).

**Figure 4:**
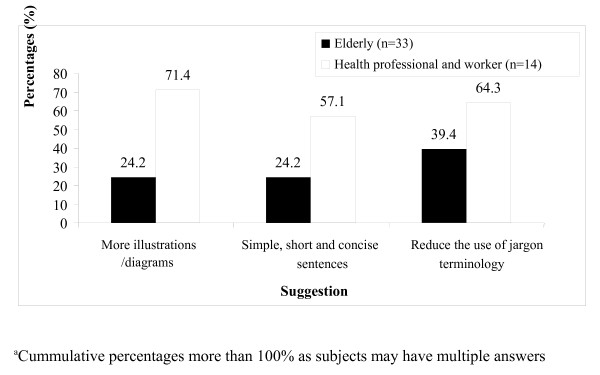
Suggestions to improve understanding of the booklet.

As shown in Figure 3, the majority (60%) of older adult subjects who did not understand the content in the booklet reported that terminology (60%), followed by illustrations (20%) and nutrition recommendations (20%) were the aspects that prevented them from understanding. Only 10% of them indicated that the sentences and tables in the booklet made it difficult to understand. Thus, investigators sought suggestions from subjects on ways to improve understanding or comprehension of the booklet, as shown in Figure 4. Suggestions provided by health staff included adding more illustrations or diagrams (71.4%), minimising uncommon terminology (64.3%) and using simpler and more concise sentences (57.1%). Older adult subjects (39.4%) suggested that use of medical terms such as 'diabetes mellitus' and 'metabolic syndrome' needed to be minimised or explained further.

## Discussion

A research-based nutrition education package on healthy ageing and reducing risk of metabolic syndrome was developed and well accepted among subjects in this study. To address the increasing prevalence of obesity and chronic diseases among Malaysian populations [1,2,19,20], the package consisted of a flipchart, placemats and a booklet. The flipchart has been developed as a teaching tool for health professionals involved in the care of older adults, particularly those living in rural areas, with the aim of reducing the risk of chronic diseases. The design of the flipchart, equipped with nutrition educator notes, was useful for staff at health clinics where there were no nutritionists or dietitians. The flipchart was also equipped with illustrations that facilitated the understanding of specific concepts such as obesity, diabetes mellitus and hyperlipidaemia. In rural areas of Malaysia where the illiteracy rate is high, counselling sessions with health professionals are the most appropriate strategy for imparting nutrition education to older adults with health and nutritional problems [21]. Individuals with low literacy often have unsatisfactory health status compared with those with higher educational level. Use of photos and illustrations can increase the understanding of health messages. In this study, most subjects understood the nutrition guidelines or messages primarily because of the illustrations provided. Print materials including a booklet and placemats developed in this study can be valuable tools for patient education, but they are only supplements and can never be a substitute for verbal communication. When used effectively, print materials can enable patients to manage their health better and help healthcare professionals maximize limited teaching time. According to the Nova Scotia Cancer Patient Education Committee [8], print materials can convey basic repetitive information, freeing the health professional to concentrate on individualized follow-up instruction. They can also facilitate consistency in teaching.

Results of the study revealed that older adult subjects had difficulty understanding the food pyramid concept compared with the food plate, as reported in the needs assessment phase [10]. In contrast, health professionals preferred the food pyramid rather than the food plate, probably because they had been trained with the pyramid. Most of the older adult subjects were exposed to food pyramids during their visits to the health clinics. The food plate concept is being used to translate nutrient recommendations to food choices in the National Food Guide in other countries [22]. Consumers prefer the food plate with respect to its visual and contextual impact compared with the food pyramid, which is deemed to be less effective in conveying messages on portion size and a balanced diet [23].

The food plate concept introduced in this study provided detailed recommendations on portion size and number of servings from the major food groups, namely cereals and grains; meat and dairy products; and fruits and vegetables, with the intake of simple sugars, fats and oils minimised. In addition, a recommendation of six to eight glasses of water a day was included because of the importance of water and higher risk of dehydration among older adults [18]. This is in agreement with fluid recommendations in Malaysian Dietary Guidelines [12]. However, until recently this recommendation was not incorporated into the Malaysian food pyramid [12].

The Dietary Guidelines for Older People in this study were delivered as food-based recommendations as opposed to the Nutrition Guide for Elderly People developed by the Ministry of Health of Malaysia, which uses a nutrient-based approach [9]. Food-based guidelines are more effective in conveying nutrition and health messages to the population as well as promoting traditional and healthy food [24].

The Malaysian Dietary Guidelines [12] were developed for the Malaysian population aged 2-60 years old. This study successfully developed dietary guidelines specific to Malaysian older adults aged 60 years and over termed ‘10 Healthy Eating Guidelines for Elderly People’. The aim of these guidelines is to provide recommendations enabling older adults to engage in a healthy lifestyle leading to a good quality of life.

The acceptance analysis of the nutrition education package showed that the majority of older adults did not understand terms such as 'diabetes mellitus', 'hypertension' or 'fibre'. In addition, they suggested the use of simple and short sentences. Researchers modified the package according to the suggestions and tested for face and content validity. However, some medical and scientific terms were retained with the intention of educating the population, with explicit explanations provided in simple language. The suggestion by health staff to incorporate more photos and illustrations in the flipchart and booklet are in agreement with the finding by Goldberg and Owen [25] that photos and illustrations should facilitate understanding of health messages without the need for text. Use of colour in developing an education package is essential in attracting interest of the target group [26]. Furthermore, the font size used in this study was considered acceptable by subjects. Small font sizes are unsuitable for older adults because of the high prevalence of eyesight problems due to ageing [27].

The intervention package can be used by health professionals and non-governmental organisations (NGOs) as a guideline and tool in the care of older adults. It is being translated into other languages such as English and Mandarin to increase its outreach among other ethnic groups in Malaysia.

## Conclusion

The nutritional education intervention package we developed was well accepted by both older adult subjects and health staff. However, modifications with respect to medical terminology and addition of more illustrations were needed to further improve the understanding and acceptability of the package. The intervention package has the potential to increase the nutrition and health knowledge of older adults and motivate them to adopt healthy eating and a healthy lifestyle, thus reducing morbidity risk and health care costs. However, health staff must be trained to use and implement the package to ensure its sustainability in improving health outcomes of older adults in the community.

## Competing interests

The authors declare that there are no competing interests.

## Authors’ contributions

SS was the project leader and responsible for the overall project and writing of the manuscript. SNA was a doctoral student involved in data collection and analysis and was involved in the writing of the manuscript. SAR acted as co-supervisor of the doctoral student and was involved in data collection and design of the education package. NY, ZY, FA and NI were co-researchers who assisted in data collection and design of the education package and results interpretation. MS was the co-researcher from the Ministry of Health involved in study design and data interpretation. AA was a researcher in anthropology who assisted in the study design and illustration of the education package and edited the manuscript. All authors read and approved the final manuscript.

## Pre-publication history

The pre-publication history for this paper can be accessed here:

http://www.biomedcentral.com/1471-2318/12/24/prepub
